# Secondary γ Transitions in ^159^*Gd* After Neutron Capture at Isolated Resonances

**DOI:** 10.6028/jres.105.024

**Published:** 2000-02-01

**Authors:** S. Posṕišil, F. Bečvář, C. Granja Bustamante, J. Kubašta, S. A. Telezhnikov

**Affiliations:** Department of Physics, Faculty of Nuclear Sciences and Physical Engineering Czech Technical University, Brehova 7, 115 19 Prague 1, Czech Republic; Department of Low Temperature Physics, Faculty of Mathematics and Physics, Charles University, V Holesovickach 2, 180 00 Prague 8, Czech Republic; Department of Physics, Faculty of Nuclear Sciences and Physical Engineering Czech Technical University, Brehova 7, 115 19 Prague 1, Czech Republic; Frank Laboratory of Neutron Physics, Joint Institute for Nuclear Research, 141 980 Dubna, Russia

**Keywords:** branching ratio, capture, energy-level transitions, energy levels, gadolinium 159, gadolinium 158 target, gamma cascades, neutron reactions, resonance neutrons

## Abstract

The ^158^Gd(n,γ)^159^Gd reaction was studied at 12 isolated neutron resonances by the TOF method at the IBR-30 Fast Pulse Reactor at JINR Dubna. Totally 15 secondary γ transitions in ^159^Gd were recorded in the range from 450 keV to 750 keV. Of these, six previously unseen transitions were placed on the established ^159^Gd level scheme. The depopulation of strongly populated levels at 507.7 keV and 558.2 keV (the head and the first excited members of band 1/2^−^ [521]) was observed for the first time. It was shown that the observed 507.7 keV γ line, masked by the annihilation peak, originates from an unresolved doublet of transitions from the 507.7 keV level to the ground state and from the 558.2 keV level to the level at 50.7 keV. The 507.7 keV level decays exclusively to the ground state, while the 558.2 keV level decays via two transitions with a branching ratio that agrees well with the prediction according to Alaga’s rule.

## 1. Introduction

The well deformed ^159^Gd nucleus is an ideal ground to test the couplings of rotational and vibrational motion to single particle modes of excitation where the unpaired nucleon (neutron) can be thought of revolving around the deformed nuclear core. While the primary γ transitions in ^159^Gd have been studied extensively from radiative neutron capture [1,2,3,4], scarce information about secondary γ transitions is known only from β^−^-decay of ^159^Eu. No (n,γ) experiment devoted to the study of secondary transitions in ^159^Gd nucleus has been done so far. Such reaction is hindered by the extremely high thermal cross sections of the neighboring isotopes, namely 155,157Gd. This work presents the data on secondary γ transitions in ^159^Gd observed following neutron capture at individual resonances of the ^158^Gd target.

## 2. Experimental

**Fig. 1:**
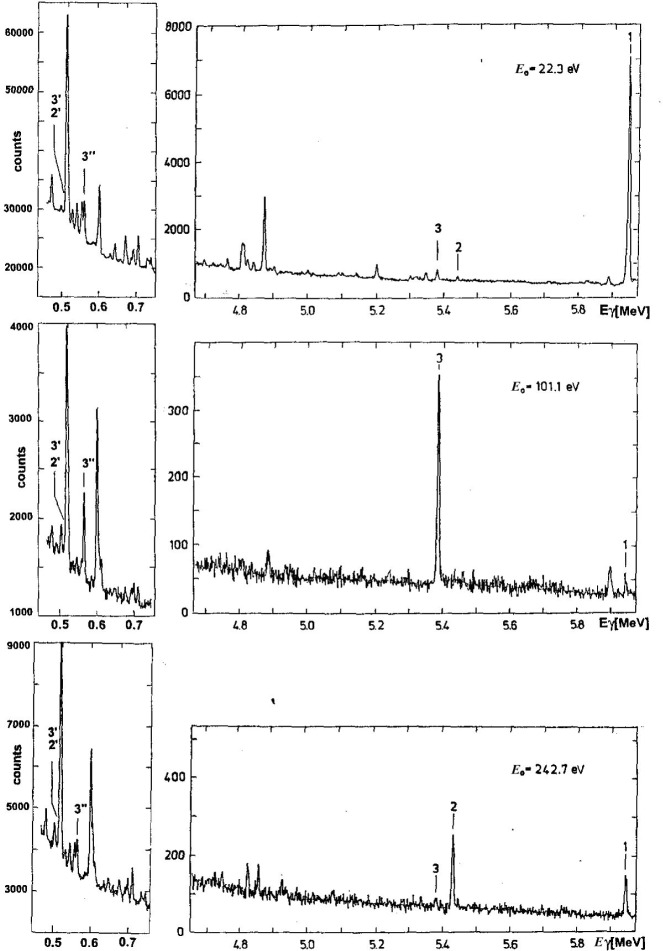
Gamma-ray spectra of ^159^Gd following neutron capture at 22.3 eV, 101.1 eV, and 242.7 eV resonances. Peaks of primary transitions to the ground state, 507.7 keV and 558.2 keV levels are denoted as 1, 2, and 3, respectively. Peaks of transitions 507.7 keV → g.s., 558.2 keV → 50.7 keV and 558.2 keV → g.s. are labelled as 2′, 3′, and 3″, respectively. Note that intensities of lines 3 and 3″ are correlated.

The experiment was carried out at the JINR Pulsed Fast Reactor IBR-30 which worked as a booster in conjunction with the 40 MeV electron linac LUE-40. The sample consisted of 48.56 g of Gd_2_O_3_ enriched in ^158^Gd to 97.7 %. The Time-Of-Flight (TOF) resolution of 70 ns/m (at 50 m flight path) enabled us to accumulate γ-ray spectra for 12 isolated neutron *s*-wave resonances with J^π^ = 1/2^+^ at energies of (22.3, 101.1, 242.7, 277.2, 344.8, 409.1, 503.3, 588.5, 692.9, 847.3, 917.1, and 1068.0) eV [5]. The resonance at energy 847.3 eV contains a small contribution of the next weak unresolved resonance at energy 869.3 eV. The γ-ray spectra, recorded by means of a Ge(Li) spectrometer, ranged from 450 keV to 750 keV and from 3.5 MeV to 6.0 MeV (neutron separation energy B_n_ = 5943.3 keV for ^159^Gd [1,3,4]). The spectra for the first three neutron resonances are shown in Fig. 1. Relative intensities of ^159^Gd γ transitions were determined in individual resonances, the total area under seven low-energy γ-lines at (467.2, 524.5, 537.1, 551.0, 601.8, 677.4 and 715.2) keV being adopted as a relative measure of number of neutrons captured at each resonance. A separate run was undertaken using a composite sample consisting of a layer of enriched Gd covered on the back side by a layer of natural boron. Comparing the yields of 477.7 keV γ rays, resulting from the ^10^B(n,αγ) reaction, and the 601.8 keV γ rays, following neutron capture in ^158^Gd at the 22.3 eV resonance, the absolute intensities of γ transitions in ^159^Gd were established. This was done by the method described in [6] omitting the role of multiple neutron scattering as the 22.3 eV resonance has a total radiative width *Γ*_γ_ significantly large compared to its neutron width *Γ*_n_. The overall uncertainty of the absolute intensity calibration in the low energy region of the spectra was estimated at 18 %.

## 3. Results and Discussion

**Fig. 2:**
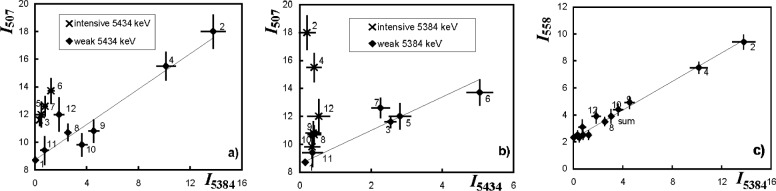
Correlations between the intensities of secondary and primary transitions. Data for the unresolved 507.7 keV doublet are shown for the primary transitions at 5384.7 keV in a) and at 5434.7 keV in b). Data points for each resonance, numbered from 1 to 12 in ascending order, are marked in a) either by “**x**” or “♦” when the 5434.7 keV transition is intense or weak, respectively. Analogous marking is used in b) to illustrate the strength of the 5384.7 keV transition. Data for the 558.1 keV and 5384.7 keV transitions are shown in c). Regression lines, calculated by least squares fit, refer to points marked by “♦”. Intensities are given in number of γ per 100 captured neutrons.

Fifteen secondary transitions were observed, of which 10 were located in the known level scheme, see Ref. [1,3,4]. Results are summarized in Table 1 where intensities are given for the strongest resonance at 22.3 eV (column 2) together with intensities averaged over the full set of 12 resonances (column 3). The unresolved γ-ray doublet at 507.7 keV, masked additionally by the neighboring 511 keV annihilation peak, was decomposed into its components belonging to the transitions 558.2 keV → 50.7 keV and 507.7 keV → g.s. This was possible thanks to correlations observed among resonances of the intensities of the 5384.7 keV primary transition, which populates the 558.2 keV level, with the intensities of the 507.7 keV γ-ray line (Fig. 2a) as well as between the intensities of the 5434.7 keV primary transition, populating the 507.7 keV level, with the intensities of the same 507.7 keV γ-ray line (Fig. 2b). The strong correlation observed between intensities of the 5384.7 keV primary transition with the intensities of the 558.1 keV secondary transition are shown in Fig. 2c. By analysing these correlations we established that the 558.2 keV level with J^π^ = 3/2^−^ decays via two transitions to the 50.7 keV level (51 %) and to the ground state (49 %). We also established that the 507.7 keV level with J^π^ = 1/2^−^ decays exclusively to the ground state. By examining the depopulation of the 558.2 keV level (Fig. 2a and 2c) the γ-branching ratio of the 558.2 keV level was determined to be equal 1.38 ± 0.26. This agrees with the branching ratio of 1.5 following from Alaga’s rule [7].

## Figures and Tables

**Table 1:** Secondary γ transitions in ^159^Gd observed at isolated neutron resonances

*E*_γ_ [keV]	*I*_γ_ [γ per 100 n]	〈*I*_γ_〉 [γ per 100 n]	*E*_f_ [keV]	→	*E*_f’_ [keV]^d^
467.2^a^ ± 0.2	1.58 ± 0.08	1.70 ± 0.06	973.7	→	507.7^c^
507.7^a^ ± 0.4	5.59 ± 0.40	6.15 ± 0.30	507.7	→	0.0^b^
507.7^a^ ± 0.4	2.36 ± 0.20	3.61 ± 0.20	558.2	→	50.7^b^
524.5 ± 0.3	0.95 ± 0.06	0.87 ± 0.04			
537.1 ± 0.2	1.62 ± 0.07	1.62 ± 0.03			
551.0 ± 0.2	2.05 ± 0.07	1.99 ± 0.03	602.1	→	50.7^c^
558.1^a^ ± 0.2	2.28 ± 0.07	3.47 ± 0.09	558.2	→	0.0^b^
601.8 ± 0.1	4.30 ± 0.05	4.34 ± 0.04	602.1	→	0.0^c^
646.9^a^ ± 0.3	1.00 ± 0.10	0.50 ± 0.20	647.2	→	0.0^c^
677.4 ± 0.3	1.88 ± 0.70	1.77 ± 0.06	744.4	→	67.8^c^
682.2 ± 0.4	0.60 ± 0.10	0.40 ± 0.10	732.6	→	50.7^c^
700.3 ± 0.4	0.60 ± 0.10	0.90 ± 0.20			
715.2^a^ ± 0.2	2.08 ± 0.08	2.33 ± 0.10	781.8	→	67.8^c^
742.4 ± 0.5	0.70 ± 0.10	0.60 ± 0.10			
747.5 ± 0.6	0.50 ± 0.10	0.20 ± 0.10			

a Newly observed γ-transitions.

b Placements based on correlations between primary and secondary γ-transitions.

c Placements based on Level-Fit calculations.

d Taken from [1,4].
